# Potential of Inactivated *Bifidobacterium* Strain in Attenuating Benzo(A)Pyrene Exposure-Induced Damage in Colon Epithelial Cells In Vitro

**DOI:** 10.3390/toxics8010012

**Published:** 2020-02-11

**Authors:** Mengfan Xu, Lili Fu, Junwen Zhang, Tao Wang, Junfeng Fan, Baoqing Zhu, Piotr Dziugan, Bolin Zhang, Hongfei Zhao

**Affiliations:** 1 College of Biological Science & Biotechnology, Beijing Forestry University, Beijing 100083, China; xumengfan1234@163.com (M.X.); sharon940329@163.com (L.F.); fanjunfeng@bjfu.edu.cn (J.F.); zhubaoqing@bjfu.edu.cn (B.Z.); 2 Beijing Key Laboratory of Forest Food Processing and Safety, Beijing Forestry University, Beijing 100083, China; 3 Institute of Fermentation Technology & Microbiology, Technical University of Lodz, 90924 Lodz, Poland

**Keywords:** *Bifidobacterium animalis* subsp. *lactis* BI-04, benzo(a)pyrene, colon epithelial cells, PI3K/AKT, CYP1A1

## Abstract

Long-term exposure to benzo(a)pyrene (BaP) poses a serious genotoxic threat to human beings. This in vitro study investigated the potential of inactivated *Bifidobacterium animalis* subsp. *lactis* BI-04 in alleviating the damage caused by BaP in colon epithelial cells. A concentration of BaP higher than 50 μM strongly inhibited the growth of colon epithelial cells. The colon epithelial cells were treated with 50 μM BaP in the presence or absence of inactivated strain BI-04 (~5 × 10^8^ CFU/mL). The BaP-induced apoptosis of the colon epithelial cells was retarded in the presence of *B. lactis* BI-04 through activation of the PI3K/ AKT signaling pathway, and p53 gene expression was decreased. The presence of the BI-04 strain reduced the intracellular oxidative stress and DNA damage incurred in the colon epithelial cells by BaP treatment due to the enhanced expression of antioxidant enzymes and metabolism-related enzymes (CYP1A1). The data from comet assay, qRT-PCR, and western blot analysis showed that the cytotoxic effects of BaP on colon epithelial cells were largely alleviated because the bifidobacterial strain could bind to this carcinogenic compound. The in vitro study highlights that the consumption of commercial probiotic strain BI-04 might be a promising strategy to mitigate BaP cytotoxicity.

## 1. Introduction

Benzo(a)pyrene (BaP), one of the polycyclic aromatic hydrocarbons, is listed as the first carcinogenic compound [1]. It is a by-product of incomplete combustion or pyrolysis of organic matter under high temperature and anoxic conditions. BaP can enter the human body through water, food, air, and other environmental vehicles [2]. BaP is highly fat-soluble, and is easily absorbed in the intestines of mammals [3]. Long-term contact with BaP can cause various types of cancers, such as lung cancer, stomach cancer, bladder cancer, and digestive tract cancer through its teratogenic and mutagenic effects [4,5,6]. Normally, BaP entering into the body is first metabolized by cytochrome P450 (CYP1A1, Cytochrome P450 Family 1 Subfamily A Member 1) to release the ultimate carcinogen 7,8-dihydroxy-9,10-epoxy-BaP (BPDE), causing cancers [6,7]. Therefore, the expression of CYP1A1 is often used as an indicator of the extent of BaP damage [8]. Varying effects of BaP against the cells have been reported. Qian et al. [9] used *Oryzias melastigma* as an in vivo model, and medaka liver cell line DIT-29 as an in vitro model to investigate the damage caused by BaP in the cells, and indicated that BaP exposure inhibited NF-κB pathway by generating sustained physiological concentrations of reactive oxygen species (ROS). The BaP treatment-induced cell injuries were also verified at the transcriptomic level [9].

Lactic acid bacteria (LAB) are gram-positive bacteria that produce lactic acid as a major fermentation metabolite. The probiotic functions of LAB have been documented in numerous studies, including the improvement of nutritional values of food and feed, stimulation of the synthesis of vitamins in the body and secretion of related enzymes, and balance of beneficial and harmful microflora in the intestine [10]. Recent studies have shown that bifidobacteria can effectively remove mycotoxin-like carcinogens [11,12,13]. In one of the recent studies, several bifidobacterial strains displayed a higher capacity of BaP adsorption. The BaP-adsorbing rate was 75.95% for *Bifidobacterium animalis* subsp. *lactis* BI-04, 74.42% for *B. animalis* subsp. *lactis* HN019, and 72.12% for *B. longum* subsp. *infantis* BY12 at 37 °C incubation for 4 h [14]. Discovering physical and chemical methods for the removal of BaP are attractive to food producers, but to date, no investigations have been carried out on the role of bifidobacterial strains in removing BaP from the surroundings, and thus, alleviating the damage of this chemical in the cell lines. Thus, the present study was designed to examine the potential application of strain BI-04 in the removal of BaP at a cellular level, especially how this strain can alleviate the BaP induced damage in colon epithelial cells was evaluated at a level of transcriptome sequencing of RNA.

## 2. Materials and Methods

### 2.1. Bacterial Strains and Culture Conditions

The probiotic strain *B. animalis* subsp. *lactis* BI-04, obtained from DuPont Co., Ltd. (Shanghai, China) was used in this study. Freeze-dried powder of this strain was cultured in MRS broth containing 0.5% L-cysteine (Sigma-Aldrich, St. Louis, MO, USA) for 24 h at 37 °C under anaerobic conditions. After sub-culturing three times, the activated bacterial cells were collected by centrifugation (4 °C, 6000× *g*, 5 min) and washed twice with sterile saline. The collected bacterial cells (approx. 5 × 10^8^ CFU/mL) were resuspended in sterile saline, and inactivated by heating (121 °C, 15 min) prior to use.

### 2.2. Preparation of Colon Epithelial Cell Culture

The human epithelial colorectal adenocarcinoma cell line Caco-2 was provided by Peking Union Medical College (Beijing, China), and the cells were maintained in MEM (Gibco, Grand Island, NY, USA) supplemented with 10% fetal bovine serum, 2% L-glutamine, 1% nonessential amino acids, 0.05 g/L penicillin, and 0.1 g/L streptomycin (Invitrogen, Carlsbad, CA, USA). Colon epithelial cells were incubated at 37 °C for 2 days in a humidified atmosphere of 5% CO_2_ until they formed monolayers. The experiments were carried out once the colon epithelial cells became differentiated.

#### 2.2.1. Preparation of BaP Working Solution

A 100 μg/mL BaP stock solution was first prepared by adding 10 mg BaP (Sigma, CA, USA) to 100 mL dimethyl sulfoxide (DMSO; Sigma). Then the BaP stock solution was diluted to *concentrations of 0, 12.5, 25, 50, and 100 μg/mL as working solutions for Thiazolyl Blue Tetrazolium Bromide (MTT) assay. The BaP working solutions were refrigerated at 4 °C for light-free storage before use.

#### 2.2.2. Determination of BaP Concentration Affecting the Growth of Colon Epithelial Cells

MTT solution at a concentration of 5 mg/mL was made by dissolving 0.5 g MTT in 100 mL phosphate-buffered saline (PBS; pH 7.4). The MTT solution was filtered through a 0.22 μm membrane filter for the removal of bacteria and was stored at 4 °C to avoid light. Colon epithelial cells were seeded into 96-well plates at a density of 3 × 10^5^ cells/mL and were incubated in a humidified atmosphere of 5% CO_2_ for 24 h, and then treated with the diluted BaP working solutions. After incubation for 4 h at 37 °C, the BaP-treated colon epithelial cells were mixed with 10 μL MTT and incubated at 37 °C for another 4 h. Next, 150 μL DMSO was added to the mixture and agitated for 10 min using a low speed shaking rocker to stop the MTT reaction. The absorbances of the viable cell lines capable of generating crystalline formazan with MTT were measured at 490 nm using a microplate reader (Bio-Rad, Hercules, CA, USA) when BaP concentration was increased from 0–100 μg/mL. The changes in the absorbance values show direct inhibition by different concentrations of BaP against colon epithelial cells. Growth inhibition of colon epithelial cells by BaP was calculated according to the formula:

$$\mathrm{Growth}\;\mathrm{Inhibition}\left(\%\right)=\frac{A0-\mathrm{Ai}}{A0}100$$

where A_0_ represents the absorbance of the control group; A_i_ indicates the absorbance of the treated group samples of BaP, BI-04, or BI-04+BaP.

### 2.3. Characteristics of Colon Epithelial Cells in the Presence of BaP and Strain BI-04

Firstly, colon epithelial cells were treated by *B. animalis* subsp. *lactis* BI-04 and BaP alone or their combination. These 4 treatments are listed in Table 1. The control was colon epithelial cells, which were only grown in DMSO medium. BaP group indicated that the cell lines were cultured with 50 μM BaP. BI-04 group meant that colon epithelial cells were cultivated with the dead cells of *B. animalis* subsp. *lactis* BI-04 (5 × 10^8^ CFU/mL). The BI-04+BaP group showed that colon epithelial cells were cultured in the presence of dead strain BI-04 and 50 μM BaP. Next, the growth of colon epithelial cells from 4 treatment groups was detected via their MTT reaction. Absorbance from the viable cells capable of generating crystalline formazan with MTT was measured at 490 nm using a microplate reader (Bio-Rad, Hercules, CA, USA).

### 2.4. Oxidative Stress Assay

Normally, oxidative stress assay covers the detection of superoxide dismutase (SOD) and glutathione (GSH) from the treated colon epithelial cells. The assay was performed as described by Chen et al. [15] with slight modifications. Colon epithelial cells undergoing treatment were digested with trypsin enzyme, washed twice with PBS, and collected by centrifugation (4 °C, 1000 × *g*, 5 min). The harvested colon epithelial cells were homogenized (1:10, *w/v*) in cold (4 °C) PBS. The lysed cells were centrifuged (10,000× *g*, 15 min, 4 °C) and the supernatants were used as the enzyme source. The activity of SOD (Units/mL) and content of GSH (mg/mL) for the treated colon epithelial cells were evaluated using kits (SOD assay kit, A001-3; reduced GSH assay kit, A006-1) purchased from Nanjing Jiancheng Bioengineering, Inc. (Nanjing, Jiangsu, China), according to the manufacturer’s instructions.

### 2.5. DNA Damage

DNA damage induced by BaP was detected using comet assay, according to the protocol reported by Adriana et al. [16]. The washed colon epithelial cells from 4 treatment groups were suspended in cold PBS (approx. 1 × 10^5^ cells/mL) after centrifugation. Comet assay for each treated sample was performed using a commercial kit (4250-050-K, Trevigen, Gaithersburg, MD, USA) according to the manufacturer’s instruction. The samples were stained with propidium iodide (PI, 20 μg/mL; Sigma) and analyzed under an epifluorescence microscope (Olympus BX-51, Tokyo, Japan) connected to an image analysis system (Comet Assay Software Project). Three hundred imaged comets were randomly selected from each slide, and the tail length (TL, μm), comet length (CL, μm), tail moment (TM), and olive tail moment (OTM) were measured using CASP software to evaluate the BaP-induced DNA damage [17].

### 2.6. Transcriptome Sequencing of RNA

To investigate how the strain BI-04 could reduce the damage caused by BaP at a molecular level, total RNA from each treated group of colon epithelial cells, as mentioned previously, was extracted using Trizol reagent (Invitrogen, Shanghai, China). Ribosomal RNA was removed from the RNA sample using an mRNA-only kit (Epicenter Biotechnologies, Madison, WI, USA) according to the manufacturer’s instructions. The quality and quantity of RNA were checked using an Agilent RNA 6000 nano Reagents Port 1 kit (Agilent, Santa Clara, CA, USA) with an Agilent 2100 Bioanalyzer. The mRNA was isolated from the total RNA using Sera-Mag Magnetic oligo(dT) particles, and then chemically fragmented. The sequence library was constructed according to the instruction of the ScriptSeq™ mRNA-Seq Library Preparation kit (Illumina-compatible). Samples were sequenced simultaneously in Illumina Hiseq2000 (San Diego, CA, USA). Quality reads were assembled into contigs, transcripts, and unigenes using Velvet and Oases software. The reads per kilobase of exon model per million mapped reads (RPKM) value was used to normalize transcript abundances. A 2-fold difference was considered to indicate differentially expressed genes. All libraries were aligned to the human genome using the HISAT [18]. The number of fragments overlapping each unigene was summarized using NCBI RefSeq and SwissProt databases. Gene ontology (GO) and Kyoto Encyclopedia of Genes and Genomes (KEGG) pathway analyses used the limma package functions goana and kegga. Comparisons in the numbers, types of regulated genes as well as metabolic pathways among the 4 treated colon epithelial cells groups indicated the potential of strain BI-04 in alleviating BaP damage.

### 2.7. CYP1A1 Expression of Colon Epithelial Cells

For the 4 treatment groups of colon epithelial cells, *CYP1A1* expression directly linked to BaP-induced stress was evaluated using real-time quantitative polymerase chain reaction (qRT-PCR) and western blot methods. Total RNA was isolated using Trizol reagent. Then, 3 µg total RNA was reverse-transcribed for each treatment. cDNA samples (2 µL) were analyzed by qRT-PCR using SYBR Green PCR Master Mix (Applied Biosystems, Foster City, CA, USA). *GAPDH* (glyceraldehyde-3-phosphate dehydrogenase) mRNA was co-amplified as a standard along with the *CYP1A1* transcripts. The primers for qRT-PCR were as follows.

*CYP1A1* forward: 5’-ACTTCATCCCTATTCTTCGCTACCT-3´

*CYP1A1* reverse: 5´-CGGATGTGGCCCTTCTCA-3´

*GAPDH* forward: 5´-CCATGGAGAAGGCTGGGG-3´

*GAPDH* reverse: 5´-CAAAGTTGTCATGGATGACC-3´

Gene expression was calculated using the comparative threshold cycle number for each treatment. The deviation (△△Cycle threshold, △△Ct) of the *CYP1A1* mRNA signal from the *GAPDH* mRNA signal was considered as an index of gene expression (%) [19]. Western blot analysis was conducted to investigate the protein expression of CYP1A1. The treated colon epithelial cells were collected and lysed in buffer containing 100 μg/mL PMSF and 100 μg/mL protease inhibitor (Roche, Basel, Switzerland) for 20 min on ice. Supernatants obtained by centrifugation (4 °C, 6000× *g*, 20 min) were boiled for 5 min after mixing with the loading buffer (5 times). Proteins in the cell lysates (15 mL) were separated by 15% sodium dodecyl sulfate-polyacrylamide gel electrophoresis (SDS-PAGE) that was run in the Mini-Protean system (Bio-Rad) and transferred to a nitrocellulose membrane (Millipore, Billerica, MA, USA). The membranes were blocked with Tris-buffered saline containing Tween-20 (TBST) and 3% bovine serum albumin for 30 min at 25 °C, and then incubated with primary antibody overnight at 4 °C. After washing 5 times with TBST, the blots were incubated with peroxidase-conjugated secondary antibody (1:1000) for 1 h at 20 °C, and then washed 6 times with TBST. Immunoreactivity was detected with an ECL Photon Chemiluminescence Western Blotting Detection Kit (Pulilai Co., Beijing, China). Gray value, defined by integrated optical density (IOD), was analyzed using TotalLab Quant software (Total Lab, Ltd., Newcastle upon Tyne, UK) to indicate the difference in CYP1A1 protein expression among the four treatment groups.

### 2.8. Statistical Analysis

All experiments were repeated three times. The data were analyzed using SPSS 18.0 (SPSS, Inc., Chicago, IL, USA) and Excel (2016) software, and is presented as the mean ± standard deviation. The level of significance was evaluated by one-way analysis of variance, and *p* < 0.05 was considered a significant difference.

## 3. Results

### 3.1. Growth Inhibition of Colon Epithelial Cells in Different Treatments

**Figure 1:**
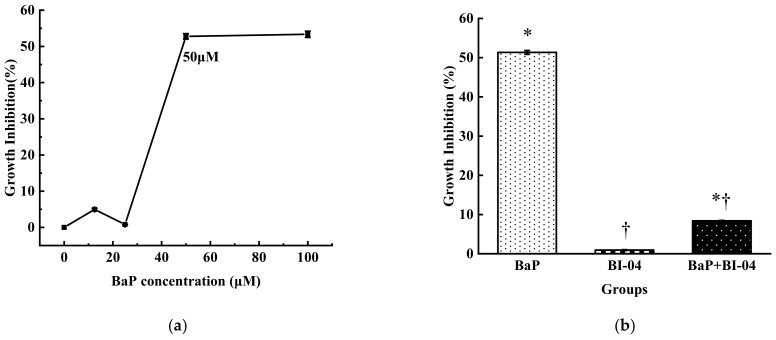
Relationship between BaP and the growth of colon epithelial cells. (**a**) Inhibition of BaP concentrations towards the growth of colon epithelial cells. (**b**) Effect of different treatments on colon epithelial cells growth. Results are expressed as mean ± standard deviation of the mean; values are mean of duplicates from three separate runs (n = 3). (* *p* < 0.05, significantly from control group, † *p* < 0.05, significantly from BaP).

No clear growth inhibition of colon epithelial cells exposed to varying concentrations of BaP 0-25 μM was observed. However, the growth of colon epithelial cells was significantly inhibited when BaP concentration reached 50 μM (Figure 1a). The treatment with more than 50 μM BaP showed significant cytotoxicity (*p* < 0.01) and incurred 52.78% growth inhibition in the colon epithelial cells. Thus, 50 μM BaP was selected for the subsequent tests. Meanwhile, MTT assay showed that the presence of *B. lactis* strain BI-04 protected colon epithelial cells from BaP-induced injuries (Figure 1b). Only 0.93% growth inhibition of colon epithelial cells was observed in the strain BI-04 group. As compared to the control, the growth inhibition of colon epithelial cells was 51.34% in the BaP treatment group, and 8.40% in the strain BI-04+BaP group. Apparently, the strain BI-04 alone did not retard the growth of colon epithelial cells but markedly relieved the damage caused by BaP in the cell lines.

### 3.2. Reaction of Oxidative Stress

**Figure 2:**
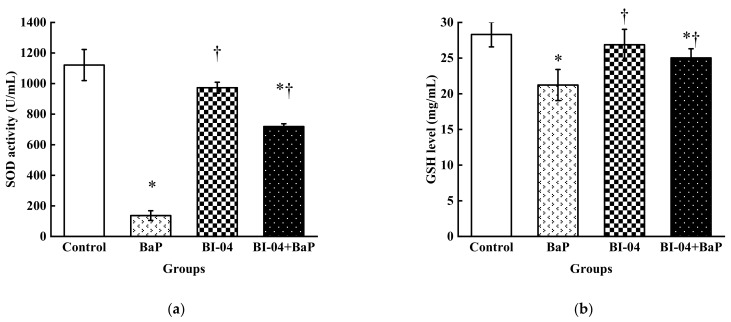
Performances of oxidative stresses of colon epithelial cells incubated at 37 °C for 4 h in different treatment groups. (**a**) represents SOD activity of colon epithelial cells and: (**b**) is the GSH level of colon epithelial cells. The results are presented as the mean ± SD of three independent assays. (* *p* < 0.05, significantly from control group, † *p* < 0.05, significantly from BaP).

As compared to the control, the SOD activity and GSH levels of colon epithelial cells, after exposure to BaP for 4 h, were markedly reduced by 87.82% and 25.01%, respectively (seen in Figure 2). On the contrary, the SOD activity and GSH levels were only slightly reduced by 13.25% and 5.09%, respectively, in the strain BI-04 group. No significant differences in the SOD activity and GSH expression levels were detected between the control and the strain BI-04 groups. The SOD activity and GSH levels of colon epithelial cells were 718.82 ± 182.98 U/mL and 25.03 ± 1.28 mg/mL, respectively in the BaP+BI-04 group. It was clear that as compared to the BaP group, the SOD activity and GSH levels of the group BaP+BI-04 were increased by 426% and 118% due to the presence of *B. lactis* BI-04. Regarding SOD activity and GSH expression levels, the oxidative injuries incurred by BaP in the colon epithelial cells was largely reduced by the probiotic *B. lactis* BI-04.

### 3.3. Analysis of DNA Damage

**Figure 3:**
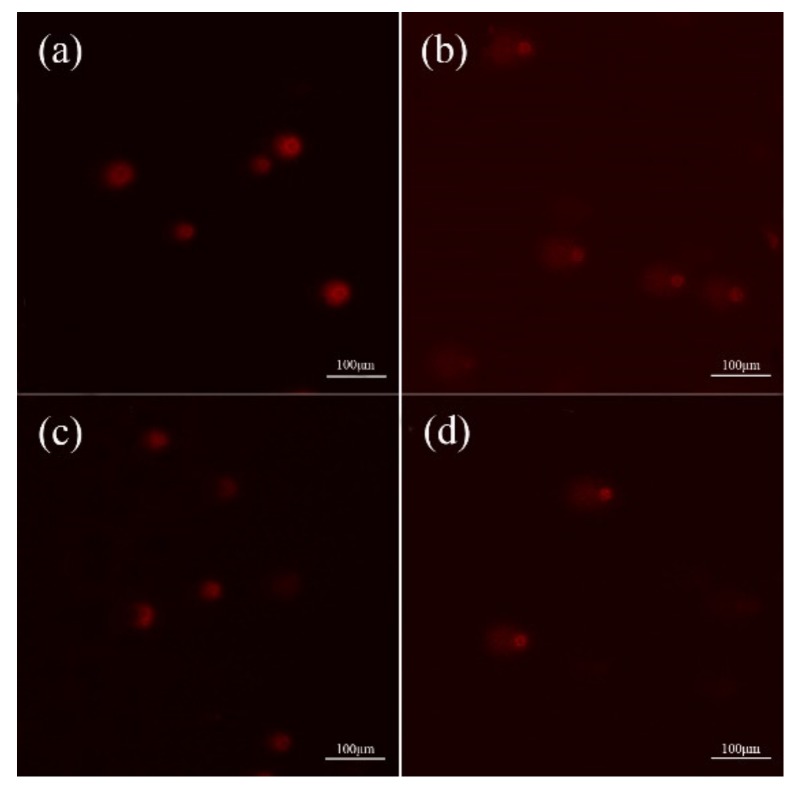
Comet assay of DNA from colon epithelial cells exposed to BaP treatments. A propidium iodide (PI) stain was done, and the images were captured under an epifluorescence microscope with a scale bar of 100 μm. (**a**) control group; (**b**) BaP treatment; (**c**) strain BI-04 group; (**d**) strain BI-04+ BaP treatment.

DNA damage of colon epithelial cells caused by BaP in four different treatment groups are presented in Figure 3 and Table 2. According to comet assay, nucleoids without DNA damage were a round-shape and bright in fluorescence intensity for the control group (Figure 3a). Fluorescence intensity of the comets decreased slightly, but no significant difference in the CL, TL, TM, and OTM were observed between the strain BI-04 group and the control colon epithelial group of cells (*p* > 0.05). These data imply that a small change in the DNA of colon epithelial cells might take place (Figure 3c and Table 2). Significantly higher DNA damage of colon epithelial cells was seen in the BaP group (Figure 3b and Table 2). The comet’s CL, TL, TM, and OTM of colon epithelial cells treated with BaP significantly increased as compared to those of the control or strain BI-04 treatment (*p* < 0.01). For the strain BI-04+BaP group, typical comet fluorescence showing DNA damage was also observed in the nucleoid colon epithelial cells. However, the extent of DNA damage was significantly lower in the strain BI-04+BaP group than the BaP treatment in terms of the comet’s CL, TL, TM, and OTM (shown in Figure 3d and Table 2) (*p* < 0.05). In other words, the injury of colon epithelial cells posed by BaP at a DNA level might be largely mitigated by the involvement of the probiotic bifidobacterial strain BI-04.

### 3.4. Transcriptomic Analysis

#### 3.4.1. Go annotation

**Figure 4:**
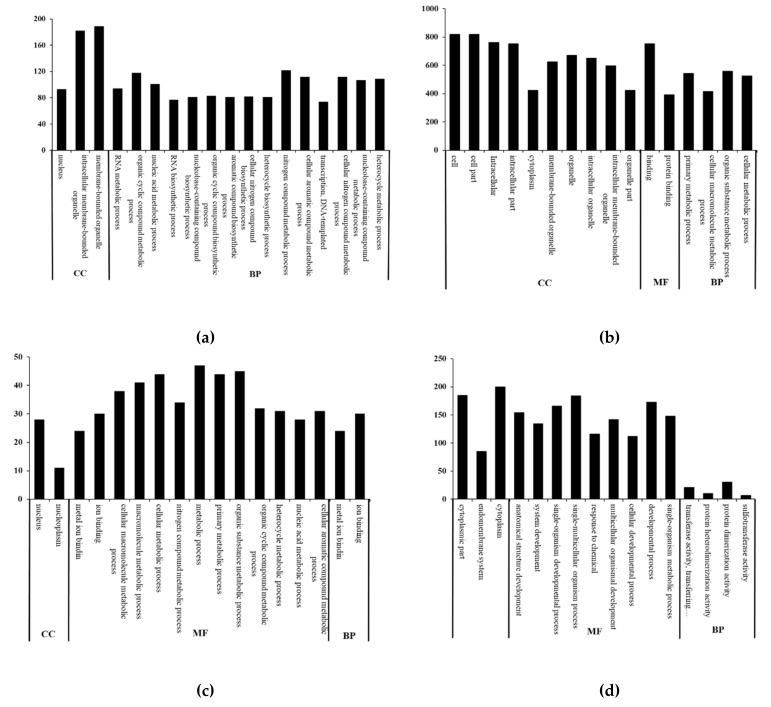
GO classification of significant differentially expressed genes (DEGs) from colon epithelial cells exposed to BaP treatments. (**a**) Control vs BI-04; (**b**) Control vs BaP; (**c**) BaP vs BI-04+BaP; (**d**) Control vs BI-04+BaP. Significant DEGs which are *p*-value ≤ 0.05 are collected. CC represents cell component; MF means molecular function; BP indicates a biological process.

Transcriptomic analysis was carried out to investigate the differences in genes and metabolic pathways of colon epithelial cells subjected to four treatments (Figure 4). As compared to the control group, 264 differentially expressed genes (DEGs) annotated from colon epithelial cells treated with strain BI-04 were linked to cell components, including 45 upregulated genes and 219 downregulated genes in the nuclei and organelles (Figure 4a). Two hundred and forty-nine annotated DEGs were attributed to biosynthesis and transcriptional regulation, including 39 upregulated genes and 210 downregulated genes (see Supplementary Tables S1 and S2). The upregulated genes were mainly responsible for the extracellular regions, transcriptional regulation, translation regulation, signal transduction, iron ions, growth factors, binding of amino compounds, cell stress response, oxidation-reduction reaction, promotion of molecular binding, and catalytic activity, and protein ubiquitination. The downregulated genes were mainly involved in mitosis, Golgi apparatus formation, the formation of membrane-free organelles, protein transport, protein phosphorylation, ATP translocase, hydrolytic enzyme, and cell adhesion. Statistically, no clear difference in the total DEGs responsible for molecular functions was found between the control and strain BI-04 groups (*p* ≤ 0.05) (see Supplementary Tables S3 and S4). Thus, despite more annotated DEGs, the strain BI-04 did not exert significant influence on the gene functions of the colon epithelial cells.

As compared to the control, 905 DEGs in the colon epithelial cells treated with BaP were identified (Figure 4b). GO annotation showed that these genes mainly related to the cellular components in the cell membranes and organelles, and included 163 upregulated genes and 742 downregulated genes. Moreover, 849 DEGs responsible for molecular functions were annotated, including 156 upregulated and 693 downregulated genes, which connect with protein binding, ion binding, and enzyme activity. A total of 861 DEGs that are linked to biosynthesis, developmental processes, and transcriptional regulation were annotated, including 155 upregulated genes and 706 downregulated genes (see Supplementary Tables S5–S7). Generally, the upregulated genes were mainly responsible for the transcription, Ca^2+^ transmembrane transport, antioxidant activity, signal transduction, cytochrome P450, DNA damage, p53 induced apoptosis, adenylate cyclase activation of G-protein coupled receptor signaling pathway, cell stress response, and organic compound metabolism. The downregulated genes were mainly involved in mitosis, epithelial cell growth and proliferation, Golgi apparatus formation, cell adhesion, cytoskeletal protein, phospholipid, and magnesium ion binding, intracellular protein transport, and hydrolytic enzyme activity (see Supplementary Tables S8–S10). Apparently, great differences between the control and BaP groups were observed in terms of the total DEGs and the gene numbers specific for molecular functions, cell components, and biological processes (*p* ≤ 0.05).

As compared to the BaP group, there were 62 DEGs responsible for nucleus and cytoplasm in the colon epithelial cells treated with strain BI-04+BaP, including 48 upregulated genes and 14 downregulated genes (Figure 4c). Fifty-seven DEGs annotated to molecular functions mainly affecting metal ion binding, out of which 44 genes were upregulated and 13 genes were downregulated. Fifty-four DEGs annotated to biological processes mainly were related to biosynthesis, including 41 upregulated and 13 downregulated genes (see Supplementary Tables S11–S13). The upregulated genes were mainly responsible for negative transcriptional regulation, zinc ion, protein, nucleotide and small molecule binding, enzyme (protein kinase, cis-trans isomerase) activity, apoptosis and cell proliferation. The down-regulated genes were mainly involved in ATP synthase complex, organelles, phospholipase activity, methyltransferase, glycosyltransferase, translation factors, mitosis, protein transport, protein phosphorylation, cell adhesion, leukocyte differentiation, long-chain fatty acid metabolism, and defense mechanisms (see Supplementary Tables S14–S16). The number of annotated DEGs, including upregulated and downregulated genes, were much lower in the colon epithelial cells treated with strain BI-04+BaP than those treated with BaP. Meanwhile, the number of annotated functional genes responsible for molecular functions, cell components, and biological process were also decreased (*p* ≤ 0.05). Interestingly, it was noted that the expression of some annotated genes, which are linked to the activity of growth metabolic enzymes and cell proliferation, were clearly enhanced due to the presence of strain BI-04, perhaps weakening the corresponding damage of BaP to colon epithelial cells (*p* ≤ 0.05). As compared to the control group, the numbers of annotated DEGs in the strain BI-04+BaP group were close to those of the strain BI-04 group. It was speculated that the involvement of strain BI-04 may help the BaP-damaged colon epithelial cells to recover a normal level (Figure 4d).

#### 3.4.2. Metabolic Pathway Analysis

The main genes and pathways in the treated colon epithelial cells are presented in Table 3. As compared to the control, 1118 differentially expressed genes (DEGs) were related to the metabolic pathways in the BaP group, and they were annotated into 290 KEGG pathways. Out of 290, 31 KEGG pathways were significant and were responsible for the metabolism and signal transduction of colon epithelial cells. A total of 80 DEGs for the strain BI-04 group were annotated into 256 KEGG pathways. Out of 256, 12 KEGG pathways were directly responsible for the metabolism and signal transduction of colon epithelial cells. Moreover, as compared to the BaP group, 80 DEGs for the strain BI-04+BaP group were attributed to metabolic pathways, and 110 were annotated to the KEGG pathways. Out of 110, 9 KEGG pathways were identified as important and were related to the metabolism and signal transduction of colon epithelial cells. Thus, it was clear that the number of the important DEG-related metabolic pathways in colon epithelial cells induced by BaP was largely reduced in the presence of the bifidobacterial strain BI-04.

### 3.5. CYP1A1 Expression in Colon Epithelial Cells

**Figure 5:**
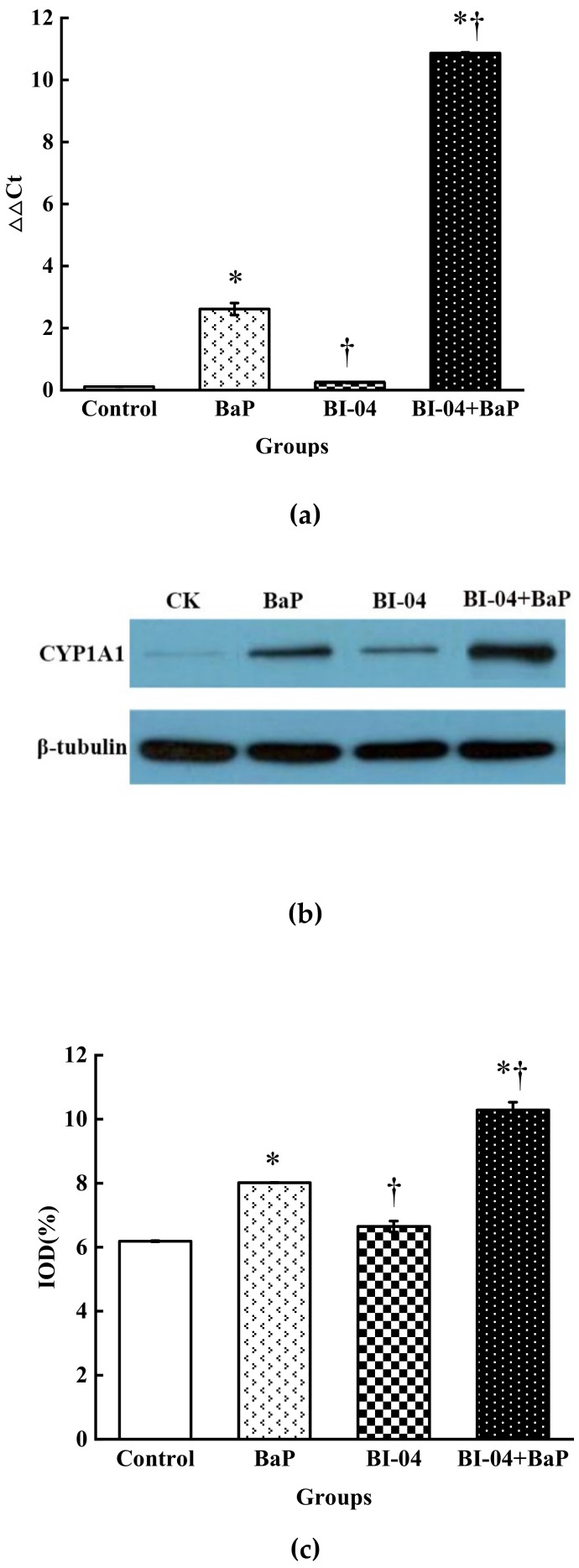
CYP1A1 expression of colon epithelial cells exposed to four BaP treatments. (**a**) *CYP1A1* mRNA expression of colon epithelial cells (△△Ct means △△Cycle threshold). (**b**) Western blot for CYP1A1 protein expression of colon epithelial cells. (**c**) IOD values of CYP1A1 from treated colon epithelial cells (IOD indicates integrated optical density). The results are presented as the mean ± SD of three independent assays. (* *p* < 0.05, significantly from CK, † *p* < 0.05, significantly from BaP).

The expression levels of *CYP1A1* mRNA and protein in colon epithelial cells from the different treatment groups were determined. As shown in Figure 5a, *CYP1A1* mRNA, determined by △△Ct, had low expression in the control and BI-04 groups. A significant increase in *CYP1A1* mRNA expression was seen in the BaP and strain BI-04+BaP groups, as compared to that in the control group (0.111 ± 0.002). A similar trend in CYP1A1 protein expression in the colon epithelial cells was observed among the four treatment groups. When β-tubulin was used as reference in the western blot analysis (Figure 5b, c), CYP1A1 expression in the colon epithelial cells was the highest in the strain BI-04+BaP group (10.287%, calculated in IOD), followed by that in the BaP group (8.017%), the strain BI-04 group (6.647%), and the control group (6.187%). The addition of BI-04 increased the CYP1A1 expression in the colon epithelial cells in the presence of BaP.

## 4. Discussion

The toxicity of BaP towards organisms has been widely reported using colon epithelial cells as an in vitro model system [20,21]. Derakhshesh et al. used MTT assay to indicate that a concentration of 100 μM BaP would lead more than 50% of the intestinal cell lines to die [22]. In this study, a concentration of 50 μM BaP was observed to strongly inhibit the growth of 52.78% colon epithelial cells, according to MTT assay. To evaluate the potential of the inactivated bifidobacterial strain in relieving BaP damage, 50 μM BaP was selected as an inhibition concentration for the following tests. Partially, oxidative stress has been regarded to be responsible for the cytotoxicity following BaP exposure, as BaP causes cellular oxidative damage by increasing the levels of free radicals in the cells [23,24]. The intracellular redox status is tightly regulated by the presence of enzymatic and non-enzymatic antioxidants, which play important roles in maintaining the free radical balance in the organisms [25]. In our study, SOD, an enzymatic antioxidant responsible for maintaining the steady status of hydrogen peroxide, and GSH, a vital non-enzymatic antioxidant that scavenges free radicals to protect the cells against oxidative stress, were investigated [15]. It was observed that colon epithelial cells treated with BaP had lower SOD activity and GSH levels as compared to the untreated controls (see Figure 2). Our results are supported by previous reports [23,26], which have shown that GSH and SOD are overutilized during the increased demand for detoxification of free radicals caused by the presence of BaP [24]. Moreover, BaP not only affected protein expression of the colon epithelial cells to a relatively high extent but also damaged their DNA and mRNA [26]. The data from comet assay indicates that the DNA of the colon epithelial cells was severely damaged by BaP. Analysis of DEGs in the control vs BaP group showed that BaP caused the colon epithelial cells to undergo apoptosis through the phosphatidylinositol 3-kinase (PI3K/AKT) pathway and MDM2/p53 pathway. The PI3K/AKT pathway is closely related to transcriptional translation and cell proliferation, growth, and apoptosis. In this study, BaP treatment downregulated the expression of genes, such as growth factor (*GF*), receptor tyrosine kinase (*RTK*), Serine/threonine protein kinase (*AKT*), I-Kappa-B Kinase (IKK), and mouse double minute 2 (*MDM2*) (see Table 3). The downregulated *GF* and *RTK* receptor, as the regional binding signal on the membrane, were transported to PI3K Ia isomer (an important signal transduction molecule in cells), catalyzing the production of the secondary messenger PIP3 on the cell membrane. Activated *AKT* regulated cell apoptosis, protein synthesis, and metabolism, and the cell cycle by phosphorylating the substrates *IKK* or *MDM2*. It is well-known that activated *p53* resists DNA damage and cell stress by causing cell cycle arrest and promoting apoptosis, and the up-regulated *p53* also induced apoptosis. Thus, *MDM2* regulated by the PI3K/AKT degraded *p53*, causing DNA damage in the colon epithelial cells. In this study, it was noted that as compared to their normal cells, the BaP treated colon epithelial cells had up-regulated expression of *CYP1A1*, *CYP1B1*, and *GST* genes, which are closely related to the metabolic pathway of aromatic hydrocarbons. The increase in the expression of CYP1A1 caused by BaP was proved by Alexandrov et al. [27]. Thus, our results indicate that BaP not only inhibits the growth of colon epithelial cells but also causes damage to the levels of DNA, mRNA, and protein in the cell lines.

To date, no investigations have been done to elucidate the potential of probiotic bifidobacterial strains or even other lactic acid bacteria in alleviating BaP-incurred damage to colon cells. In our study, four treatment conditions including control, strain BI-04, BaP, and strain BI-04+BaP were designed to evaluate the possible role of *B. animalis* subsp. *lactis* BI-04 in protecting colon epithelial cells from BaP toxicity. It was found that the presence of dead bifidobacterial strain mitigated BaP-induced cytotoxicity against colon epithelial cells. The BaP-induced growth inhibition of colon epithelial cells was relieved in the presence of *B. lactis* BI-04 at a concentration of 5×10^8^ CFU/mL. Similar results were also reported by He et al. [28]. They indicated that the degree of deformation of colon epithelial cells caused by BaP alone was decreased upon combination treatment with BaP and LAB strains. Compared to treatment with BaP, the treatment strain BI-04+BaP eliminated the toxic effects of free radicals through enhancing the expression levels of GSH and activity levels of SOD. The BaP exposure-induced damage in colon epithelial cells could be detoxified due to the presence of strain BI-04. A similar protective mechanism was reported for *Lactobacillus plantarum* CCFM639, which alleviated aluminum toxicity in the liver, kidney, and brain of mice by altering SOD activity and GSH expression levels [29]. Comparisons among four groups demonstrated that activity of the PI3K/AKT and MDM2/ p53 signaling pathways could be returned to normal levels in the presence of strain BI-04, which upregulated the levels of *PI3K* and *MDM2* gene expression significantly. The inactivated strain BI-04 might alleviate BaP cytotoxicity via increased levels of PI3K and DNA repair-related gene transcription. DNA was damaged largely by BaP treatment alone, but the addition of strain BI-04 increased the expression of CYP1A1, a key detoxification agent, which can effectively protect colon epithelial cells against BaP-induced DNA damage under in vitro conditions (See Figure 5) [30]. Similar results were reported by Uno et al. [31,32] who showed that the elevated expression of CYP1A1 in intestinal cells was beneficial for detoxification of BaP. An in vivo study showed that after oral administration of BaP, normal *Cyp1*(+/+) mice cleared blood BaP at least four-fold faster than *CYP1A1*(-/-) knockout mice [31]. Overexpression of CYP1A1 in hepatocytes directly inhibited the formation of DNA polymers, thus reducing the DNA damage induced by BaP [33]. Evidence from our comet assay indicates that compared to that by the BaP treatment alone, CL, TL, TM, and OTM of colon epithelial cells were largely alleviated by the combination treatment of strain BI-04 and BaP (See Figure 3 and Table 2). The presence of strain BI-04 might retard the formation of DNA polymers by increased expression of CYP1A1, attenuating BaP toxicity towards colon epithelial cells. Recently, several studies have explored the efficiency of flavonoids and butyrates in weakening the cytotoxicity of BaP using different cell lines. It was seen that flavonoids did not decrease AhR expression but counteracted BaP-mediated aryl hydrocarbon receptor repressor (AhRR) repression [22,34,35]. It was found that butyrate not only altered the expression of cytochrome CYP1A1 and metabolism of BaP via histone deacetylase activity but also improved the expression and activity of xenobiotic enzymes, which are responsible for metabolizing carcinogens in the colon epithelial cell model. The expression of *CYP1A1* mRNA and protein between the control and the strain BI-04 groups was also marginally different. The involvement of strain BI-04 led to increased expression of CYP1A1, thus increasing the tolerance of colon epithelial cells to BaP toxicity. Sunil et al. [36] stated that epigallocatechin gallate from white tea was beneficial as a protective agent against BaP-induced lung damage. However, few studies have clarified the mechanism of probiotic bacteria, such as strain BI-04, in alleviating BaP-induced injury against colon epithelial cells at the molecular level.

LAB strains have a high ability to adsorb mycotoxins like ochratoxin A, and AFB1/2 in the water-soluble phase. *L. acidophilus* could remove at least 95% ochratoxin A and *B. animalis* could remove 80% bra toxins [11,37,38]. In our recent investigations, both live and dead cells of *B. animalis* subsp. *lactis* BI-04 removed most of the BaP via its physical binding model [14]. Thus, a possible mechanism by which inactivated *B. animalis* subsp. *lactis* BI-04 reduces BaP toxicity towards colon epithelial cells is the physical adsorption of this probiotic microorganism to the chemical. As a result, the upregulation of PI3K/AKT pathway and CYP1A1 expression were started in the presence of strain BI-04, and the colon cell lines damage might be alleviated at the epigenetic and molecular levels.

## 5. Conclusions

The present study indicates that a concentration of 50 μM BaP can not only produce apparent inhibition of the growth of colon epithelial cells, but could also cause serious cytotoxicity, including oxidative injuries, DNA damage, and abnormal expression of *CYP1A1* and *p53*. However, the presence of strain BI-04 at a concentration of approximately 5 × 10^8^ CFU/mL can alleviate BaP-induced cytotoxicity by regulating the DEGs and metabolic pathways, promoting the anti-oxidation, scavenging the DNA damage, and inhibiting apoptosis. The ability of strain BI-04 to bind BaP might, in turn, reduce the toxicity of this chemical towards colon cell lines. To our knowledge, this is the first report addressing that *B animalis* subsp. *lactis* BI-04 may be useful in biological detoxification for removing BaP from the in vitro cell model. 

## Figures and Tables

**Table 1:** Treatments of colon epithelial cells.

Components	Groups			
	Control	BaP	BI-04	BI-04 + BaP
DMSO	0.1%	—	—	—
BaP	0μM	50μM	0μM	50μM
Bacterial suspension (5 × 10^8^ CFU/mL)	—	—	*B. lactis* BI-04	*B. lactis* BI-04

Notes. Colon epithelial cells were exposed to the different treatment groups at 37 °C for 4 h. BaP solution was prepared as in method 2.4, and the final concentration was 50 μM. Bacterial suspension was prepared as in method 2.2. All treatments were performed in triplicate.

**Table 2:** DNA damage of colon epithelial cells induced by various BaP treatments.

Groups	DNA Damage Indicators			
	Comet Length	Tail Length	Tail Moment	OliveTail Moment
Control	155.31 ± 44.94	17.00 ± 15.09	0.39 ± 0.49	1.04 ± 0.88
BaP	301.88 ± 67.97 *	143.21 ± 48.35 *	73.14 ± 31.47 *	47.75 ± 15.82 *
BI-04	160.00 ± 11.63 †	30.69 ± 21.90 *†	4.44 ± 2.89 *†	5.11 ± 2.12 *†
BI-04+BaP	284.17 ± 48.80 *†	123.67 ± 33.69 *†	47.69 ± 22.91 *†	37.66 ± 9.78 *†

Notes. Effects of BaP and inactivated *B. animalis* subsp. *lactis* BI-04 treatments on colon epithelial cells DNA were determined by comet assay. The results, expressed as Comet Length, Tail Length, Tail Moment, and Olive Tail Moment, represent mean ± standard deviation. Values are the mean of duplicates from three separate runs (n = 3). (* *p* < 0.05, significantly from the control group, †* p* < 0.05, significantly from BaP group).

**Table 3:** Main genes and pathways of treated colon epithelial cells.

Gene Regulation	Major Genes and Pathways Related to Cytotoxicity in the Control vs BaP groups	Major Genes and Pathways Linked to Cytotoxicity in the BaP vs Strain BI-04+BaP Groups
**UP**	*-p53* (MDM2/p53 signal pathway); -*CYP1A1*, *CYP1B1* and *GST* (Chemical carcinogenesis pathway)	-*CYP1A1* (Metabolism of xenobiotics by cytochrome P450 pathway); -*PI3K* (PI3K/AKT signal pathway).
**DOWN**	-*GF, RTK, AKT, IKK, MDM2*, et al. (PI3K/AKT signal pathway);	—

## Supplementary Materials

The following are available online at https://www.mdpi.com/2305-6304/8/1/12/s1, Table S1. GO classification of DEGs between Control and BI-04 (Cell Component); Table S2. GO classification of DEGs between Control and BI-04 (Biological Process), Table S3. The up-regulated genes and down-regulated genes in cell components between Control and BI-04, Table S4. The up-regulated genes and down-regulated genes in biological process between Control and BI-04, Table S5. GO classification of DEGs between Control and BaP (cell component), Table S6. GO classification of DEGs between Control and BaP (Molecar Function), Table S7. GO classification of DEGs between Control and BaP (Biological Process), Table S8. The up-regulated genes and down-regulated genes in cell components between Control and BaP, Table S9. The up-regulated genes and down-regulated genes in molecular function between Control and BaP, Table S10. The up-regulated genes and down-regulated genes in biological process between Control and BaP, Table S11. GO classification of DEGs between BaP and BI-04+BaP (Cell Component), Table S12. GO classification of DEGs between BaP and BI-04+BaP (Molecular Function), Table S13. GO classification of DEGs between BaP and BI-04+BaP (Biological Process), Table S14. The up-regulated genes and down-regulated genes in cell components between BaP and BI-04+BaP, Table S15. The up-regulated genes and down-regulated genes in molecular function between BaP and BI-04+BaP, Table S16. The up-regulated genes and down-regulated genes in biological process between BaP and BI-04+BaP.
