# Supplementary material for: Potential of Inactivated Bifidobacterium Strain in Attenuating Benzo(A)Pyrene Exposure-Induced Damage in Colon Epithelial Cells In Vitro
Source: Toxics. 2020 Feb 11;8(1):12. doi: 10.3390/toxics8010012 (PMC7151743; doi:10.3390/toxics8010012)
Supplement: Supplementary file 1 [file toxics-08-00012-s001.pdf]

# Potential of Inactivated *Bifidobacterium* Strain in Attenuating Benzo(A)Pyrene Exposure-Induced Damage in Colon Epithelial Cells In Vitro

**Mengfan Xu, Lili Fu, Junwen Zhang, Tao Wang, Junfeng Fan, Baoqing Zhu, Piotr Dziugan, Bolin Zhang and Hongfei Zhao**

**Table S1.** GO classification of DEGs between Control and BI-04（Cell Component）.

| Gene Ontology Term | Cluster Frequency | Corrected P-value |
| --- | --- | --- |
| nucleus | 93 out of 264 genes, 35.2% | 0.00042 |
| intracellular membrane-bounded organelle | 182 out of 264 genes, 68.9% | 0.00863 |
| membrane-bounded organelle | 189 out of 264 genes, 71.6% | 0.00997 |
| extracellular space | 10 out of 264 genes, 3.8% | 0.03126 |
| mitotic cohesin complex | 2 out of 264 genes, 0.8% | 0.04794 |

**Table S2.** GO classification of DEGs between Control and BI-04（Biological Process）.

| Gene Ontology Term | Cluster Frequency | Corrected P-value |
| --- | --- | --- |
| RNA metabolic process | 94 out of 249 genes, 37.8% | 0.00046 |
| organic cyclic compound metabolic process | 118 out of 249 genes, 47.4% | 0.00198 |
| nucleic acid metabolic process | 101 out of 249 genes, 40.6% | 0.00226 |
| RNA biosynthetic process | 77 out of 249 genes, 30.9% | 0.00305 |
| nucleobase-containing compound biosynthetic process | 81 out of 249 genes, 32.5% | 0.00317 |
| organic cyclic compound biosynthetic process | 83 out of 249 genes, 33.3% | 0.00408 |
| aromatic compound biosynthetic process | 81 out of 249 genes, 32.5% | 0.00488 |
| cellular nitrogen compound biosynthetic process | 82 out of 249 genes, 32.9% | 0.00541 |
| heterocycle biosynthetic process | 81 out of 249 genes, 32.5% | 0.00565 |
| nitrogen compound metabolic process | 122 out of 249 genes, 49.0% | 0.00658 |
| cellular aromatic compound metabolic process | 112 out of 249 genes, 45.0% | 0.00955 |
| transcription, DNA-templated | 74 out of 249 genes, 29.7% | 0.01589 |
| cellular nitrogen compound metabolic process | 112 out of 249 genes, 45.0% | 0.02414 |
| nucleobase-containing compound metabolic process | 107 out of 249 genes, 43.0% | 0.03089 |
| heterocycle metabolic process | 109 out of 249 genes, 43.8% | 0.0341 |

**Table S3.** The up-regulated genes and down-regulated genes in cell components between Control and BI-04.

| Up-regulated EDGs | | Down-regulated EDGs | |
| --- | --- | --- | --- |
| GO:0005615 | extracellular space | GO:0005794 | Golgi apparatus |
| GO:0005886 | plasma membrane | GO:0005813 | centrosome |
| GO:0000922 | spindle pole | GO:0000139 | Golgi membrane |

| | | | |
|---|---|---|---|
| GO:0000932 | cytoplasmic mRNA processing body | GO:0005887 | integral component of plasma membrane |
| GO:0001725 | stress fiber | GO:0030529 | intracellular ribonucleoprotein complex |
| GO:0005578 | proteinaceous extracellular matrix | GO:0043232 | intracellular non-membrane-bounded organelle |
| GO:0005681 | spliceosomal complex | | |
| GO:0005764 | lysosome | | |
| GO:0005777 | peroxisome | | |
| GO:0005787 | signal peptidase complex | | |

**Table S4.** The up-regulated genes and down-regulated genes in biological process between Control and BI-04.

| Up-regulated EDGs | | Down-regulated EDGs | |
|---|---|---|---|
| GO:0001666 | response to hypoxia | GO:0007067 | mitotic nuclear division |
| GO:0006412 | translation | GO:0007154 | Cell communication |
| GO:0009267 | cellular response to starvation | GO:0015031 | protein transport |
| GO:0009636 | response to toxic substance | GO:0006468 | protein phosphorylation |
| GO:0044093 | positive regulation of molecular function | GO:0007155 | cell adhesion |
| GO:0000209 | protein polyubiquitination | | |

**Table S5.** GO classification of DEGs between Control and BaP（cell component）.

| Gene Ontology term | Cluster frequency | Corrected P-value |
|---|---|---|
| cell | 818 out of 905 genes, 90.4% | 1.21E-11 |
| cell part | 818 out of 905 genes, 90.4% | 1.21E-11 |
| intracellular | 761 out of 905 genes, 84.1% | 7.77E-10 |
| intracellular part | 753 out of 905 genes, 83.2% | 9.92E-09 |
| nucleus | 284 out of 905 genes, 31.4% | 1.67E-07 |
| cytoplasm | 422 out of 905 genes, 46.6% | 2.36E-06 |
| endomembrane system | 177 out of 905 genes, 19.6% | 2.38E-06 |
| membrane-bounded organelle | 624 out of 905 genes, 69.0% | 2.62E-06 |
| organelle | 671 out of 905 genes, 74.1% | 4.88E-06 |
| intracellular organelle | 651 out of 905 genes, 71.9% | 7.67E-06 |
| intracellular membrane-bounded organelle | 595 out of 905 genes, 65.7% | 1.14E-05 |
| intracellular organelle part | 392 out of 905 genes, 43.3% | 0.00015 |
| cytoplasmic part | 375 out of 905 genes, 41.4% | 0.00021 |
| endoplasmic reticulum | 64 out of 905 genes, 7.1% | 0.00026 |
| organelle part | 422 out of 905 genes, 46.6% | 0.00074 |
| cell junction | 56 out of 905 genes, 6.2% | 0.0008 |
| non-membrane-bounded organelle | 219 out of 905 genes, 24.2% | 0.00083 |
| intracellular non-membrane-bounded organelle | 219 out of 905 genes, 24.2% | 0.00083 |
| nuclear part | 223 out of 905 genes, 24.6% | 0.00213 |

| | | |
|---|---|---|
| cytoskeletal part | 84 out of 905 genes, 9.3% | 0.00215 |
| cytoskeleton | 125 out of 905 genes, 13.8% | 0.0024 |
| membrane region | 49 out of 905 genes, 5.4% | 0.00266 |
| protein complex | 196 out of 905 genes, 21.7% | 0.00305 |
| intracellular organelle lumen | 211 out of 905 genes, 23.3% | 0.01002 |
| membrane-enclosed lumen | 212 out of 905 genes, 23.4% | 0.0107 |
| organelle lumen | 211 out of 905 genes, 23.3% | 0.01222 |
| cytosol | 65 out of 905 genes, 7.2% | 0.01571 |
| Golgi apparatus | 53 out of 905 genes, 5.9% | 0.01996 |
| postsynaptic membrane | 9 out of 905 genes, 1.0% | 0.02136 |
| nuclear lumen | 199 out of 905 genes, 22.0% | 0.02563 |
| chromosome | 61 out of 905 genes, 6.7% | 0.02663 |
| cell-cell junction | 29 out of 905 genes, 3.2% | 0.03436 |
| inclusion body | 9 out of 905 genes, 1.0% | 0.03531 |
| centrosome | 24 out of 905 genes, 2.7% | 0.03838 |
| microtubule cytoskeleton | 77 out of 905 genes, 8.5% | 0.03859 |
| bounding membrane of organelle | 78 out of 905 genes, 8.6% | 0.04544 |

**Table S6.** GO classification of DEGs between Control and BaP（Molecar Function）.

| Gene Ontology term | Cluster frequency | Corrected P-value |
|---|---|---|
| binding | 753 out of 849 genes, 88.7% | 2.81E-06 |
| protein binding | 392 out of 849 genes, 46.2% | 0.00022 |
| ion binding | 321 out of 849 genes, 37.8% | 0.00111 |
| enzyme binding | 98 out of 849 genes, 11.5% | 0.00165 |
| zinc ion binding | 49 out of 849 genes, 5.8% | 0.00398 |
| metal ion binding | 227 out of 849 genes, 26.7% | 0.00444 |
| DNA binding | 107 out of 849 genes, 12.6% | 0.00481 |
| sequence-specific DNA binding RNA polymerase II transcription factor activity | 31 out of 849 genes, 3.7% | 0.01864 |
| sequence-specific DNA binding transcription factor activity | 45 out of 849 genes, 5.3% | 0.01921 |
| transition metal ion binding | 156 out of 849 genes, 18.4% | 0.0249 |
| mannosyl-oligosaccharide mannosidase activity | 5 out of 849 genes, 0.6% | 0.03075 |
| protein heterodimerization activity | 14 out of 849 genes, 1.6% | 0.03937 |
| anion binding | 87 out of 849 genes, 10.2% | 0.04402 |

**Table S7.** GO classification of DEGs between Control and BaP（Biological Process）.

| Gene Ontology term | Cluster frequency | Corrected P-value |
|---|---|---|
| nervous system development | 131 out of 861 genes, 15.2% | 2.02E-06 |
| regulation of cellular macromolecule biosynthetic process | 113 out of 861 genes, 13.1% | 1.22E-05 |
| multicellular organismal development | 265 out of 861 genes, 30.8% | 1.93E-05 |
| regulation of transcription, DNA-templated | 104 out of 861 genes, 12.1% | 2.46E-05 |
| regulation of nitrogen compound metabolic process | 140 out of 861 genes, 16.3% | 3.49E-05 |
| regulation of nucleobase-containing compound metabolic process | 138 out of 861 genes, 16.0% | 3.93E-05 |
| cardiovascular system development | 68 out of 861 genes, 7.9% | 4.38E-05 |

| | | |
|---|---|---|
| circulatory system development | 68 out of 861 genes, 7.9% | 4.38E-05 |
| system development | 241 out of 861 genes, 28.0% | 5.03E-05 |
| single-organism developmental process | 305 out of 861 genes, 35.4% | 8.00E-05 |
| regulation of RNA biosynthetic process | 105 out of 861 genes, 12.2% | 9.04E-05 |
| anatomical structure development | 278 out of 861 genes, 32.3% | 0.00015 |
| regulation of RNA metabolic process | 106 out of 861 genes, 12.3% | 0.00015 |
| regulation of macromolecule biosynthetic process | 121 out of 861 genes, 14.1% | 0.00016 |
| regulation of biosynthetic process | 127 out of 861 genes, 14.8% | 0.00027 |
| positive regulation of cellular process | 163 out of 861 genes, 18.9% | 0.00031 |
| cell differentiation | 175 out of 861 genes, 20.3% | 0.00047 |
| organic substance biosynthetic process | 311 out of 861 genes, 36.1% | 0.00056 |
| regulation of cellular biosynthetic process | 119 out of 861 genes, 13.8% | 0.00063 |
| neuron differentiation | 65 out of 861 genes, 7.5% | 0.00064 |
| biosynthetic process | 315 out of 861 genes, 36.6% | 0.00088 |
| macromolecule biosynthetic process | 273 out of 861 genes, 31.7% | 0.00091 |
| regulation of cellular metabolic process | 200 out of 861 genes, 23.2% | 0.00098 |
| cell development | 86 out of 861 genes, 10.0% | 0.00104 |
| primary metabolic process | 543 out of 861 genes, 63.1% | 0.00134 |
| positive regulation of cellular metabolic process | 97 out of 861 genes, 11.3% | 0.00153 |
| cellular macromolecule biosynthetic process | 268 out of 861 genes, 31.1% | 0.00154 |
| negative regulation of cell differentiation | 34 out of 861 genes, 3.9% | 0.00155 |
| response to endogenous stimulus | 87 out of 861 genes, 10.1% | 0.00176 |
| positive regulation of metabolic process | 106 out of 861 genes, 12.3% | 0.00184 |
| anatomical structure morphogenesis | 143 out of 861 genes, 16.6% | 0.00197 |
| transcription, DNA-templated | 216 out of 861 genes, 25.1% | 0.002 |
| generation of neurons | 78 out of 861 genes, 9.1% | 0.00244 |
| negative regulation of developmental process | 38 out of 861 genes, 4.4% | 0.00271 |
| RNA biosynthetic process | 217 out of 861 genes, 25.2% | 0.00275 |
| regulation of primary metabolic process | 189 out of 861 genes, 22.0% | 0.00304 |
| cellular biosynthetic process | 301 out of 861 genes, 35.0% | 0.00325 |
| cellular macromolecule metabolic process | 415 out of 861 genes, 48.2% | 0.00345 |
| single-multicellular organism process | 332 out of 861 genes, 38.6% | 0.00355 |
| regulation of metabolic process (view genes) | 230 out of 861 genes, 26.7% | 0.00415 |
| neurogenesis | 81 out of 861 genes, 9.4% | 0.00432 |
| developmental process | 317 out of 861 genes, 36.8% | 0.00433 |
| cellular developmental process | 194 out of 861 genes, 22.5% | 0.00472 |
| regulation of cell differentiation | 63 out of 861 genes, 7.3% | 0.0052 |
| protein modification process | 171 out of 861 genes, 19.9% | 0.00613 |
| cell cycle | 110 out of 861 genes, 12.8% | 0.0065 |
| organic substance metabolic process | 559 out of 861 genes, 64.9% | 0.00685 |
| macromolecule modification | 177 out of 861 genes, 20.6% | 0.00894 |
| cellular protein modification process | 168 out of 861 genes, 19.5% | 0.00974 |
| regulation of macromolecule metabolic process | 182 out of 861 genes, 21.1% | 0.01018 |
| organic cyclic compound biosynthetic process | 234 out of 861 genes, 27.2% | 0.01179 |
| negative regulation of cellular process | 132 out of 861 genes, 15.3% | 0.01228 |
| positive regulation of gene expression | 41 out of 861 genes, 4.8% | 0.01263 |

| | | |
|---|---|---|
| positive regulation of nitrogen compound metabolic process | 47 out of 861 genes, 5.5% | 0.01325 |
| cellular component organization | 266 out of 861 genes, 30.9% | 0.01638 |
| cell cycle process | 96 out of 861 genes, 11.1% | 0.01677 |
| positive regulation of biological process | 182 out of 861 genes, 21.1% | 0.01906 |
| positive regulation of nucleobase-containing compound metabolic process | 46 out of 861 genes, 5.3% | 0.01986 |
| vasculature development | 44 out of 861 genes, 5.1% | 0.02144 |
| positive regulation of transcription, DNA-templated | 38 out of 861 genes, 4.4% | 0.02295 |
| cellular component morphogenesis | 62 out of 861 genes, 7.2% | 0.02363 |
| positive regulation of macromolecule metabolic process | 88 out of 861 genes, 10.2% | 0.02569 |
| regulation of transcription from RNA polymerase II promoter | 51 out of 861 genes, 5.9% | 0.0279 |
| cellular component organization or biogenesis | 272 out of 861 genes, 31.6% | 0.02807 |
| cellular metabolic process | 526 out of 861 genes, 61.1% | 0.02819 |
| nucleic acid metabolic process | 287 out of 861 genes, 33.3% | 0.02979 |
| positive regulation of RNA biosynthetic process | 38 out of 861 genes, 4.4% | 0.0386 |
| nucleobase-containing compound biosynthetic process | 223 out of 861 genes, 25.9% | 0.04117 |
| aromatic compound biosynthetic process | 225 out of 861 genes, 26.1% | 0.04186 |
| axon guidance | 18 out of 861 genes, 2.1% | 0.04192 |
| neuron projection guidance | 18 out of 861 genes, 2.1% | 0.04192 |

**Table S8.** The up-regulated genes and down-regulated genes in cell components between Control and BaP.

| Up-regulated EDGs | | Down-regulated EDGs | |
|---|---|---|---|
| GO:0000932 | cytoplasmic mRNA processing body | GO:0005813 | centrosome |
| GO:0005669 | transcription factor TFIID complex | GO:0005794 | Golgi apparatus |
| GO:0005776 | autophagosome | GO:0097060 | synaptic membrane |
| GO:0005844 | polysome | GO:0030054 | cell junction |
| GO:0005849 | mRNA cleavage factor complex | GO:0005764 | lysosome |
| GO:0005891 | voltage-gated calcium channel complex | GO:0005769 | early endosome |
| GO:0031074 | nucleocytoplasmic shuttling complex | GO:0016323 | basolateral plasma membrane |
| GO:0031201 | SNARE complex | GO:0031965 | nuclear membrane |
| GO:0031225 | anchored component of membrane | | |
| GO:0034704 | calcium channel complex | | |

**Table S9.** The up-regulated genes and down-regulated genes in molecular function between Control and BaP.

| Up-regulated EDGs | | Down-regulated EDGs | |
|---|---|---|---|

| | | | |
|---|---|---|---|
| GO:0016209 | antioxidant activity | GO:0008092 | cytoskeletal protein binding |
| GO:0003729 | mRNA binding | GO:0005543 | phospholipid binding |
| GO:000495 | dopamine neurotransmitter receptor activity | GO:0017016 | Ras GTPase binding |
| GO:0008106 | alcohol dehydrogenase (NADP+) activity | GO:0003774 | motor activity |
| GO:0008408 | 3'-5' exonuclease activity | GO:0005509 | calcium ion binding |
| GO:0009055 | electron carrier activity | GO:0005539 | glycosaminoglycan binding |
| GO:0003684 | damaged DNA binding | GO:0000287 | magnesium ion binding |
| GO:0005035 | death receptor activity | | |

**Table S10.** The up-regulated genes and down-regulated genes in biological process between Control and BaP.

| Up-regulated EDGs | | Down-regulated EDGs | |
|---|---|---|---|
| GO:0001523 | retinoid metabolic process | GO:0007599 | hemostasis |
| GO:0007189 | adenylate cyclase-activating G-protein coupled receptor signaling pathway | GO:0007067 | mitotic nuclear division |
| GO:0009267 | cellular response to starvation | GO:0019538 | protein metabolic process |
| GO:0009812 | flavonoid metabolic process | GO:0000087 | mitotic M phase |
| GO:0030647 | aminoglycoside antibiotic metabolic process | GO:0006886 | intracellular protein transport |
| GO:0000060 | protein import into nucleus, translocation | GO:0019827 | stem cell population maintenance |
| GO:0048167 | regulation of synaptic plasticity | GO:0006928 | movement of cell or subcellular component |
| GO:0006304 | DNA modification | GO:0016337 | single organismal cell-cell adhesion |
| GO:0009753 | response to jasmonic acid | GO:0040007 | growth |
| GO:0072331 | signal transduction by p53 class mediator | GO:0001935 | endothelial cell proliferation |

**Table S11.** GO classification of DEGs between BaP and BI-04+BaP（Cell Component）.

| Gene Ontology term | Cluster frequency | Corrected P-value |
|---|---|---|
| nucleus | 28 out of 62 genes, 45.2% | 0.00686 |
| nucleoplasm | 11 out of 62 genes, 17.7% | 0.02477 |

**Table S12.** GO classification of DEGs between BaP and BI-04+BaP（Molecular Function）.

| Gene Ontology term | Cluster frequency | Corrected P-value |
|---|---|---|

| | | |
|---|---|---|
| metal ion bindin | 24 out of 57 genes, 42.1% | 0.01861 |
| ion binding | 30 out of 57 genes, 52.6% | 0.03843 |

**Table 13.** GO classification of DEGs between BaP and BI-04+BaP（Biological Process）.

| Gene Ontology term | Cluster frequency | Corrected P-value |
|---|---|---|
| cellular macromolecule metabolic process | 38 out of 54 genes, 70.4% | 0.00381 |
| macromolecule metabolic process | 41 out of 54 genes, 75.9% | 0.00484 |
| cellular metabolic process | 44 out of 54 genes, 81.5% | 0.0108 |
| nitrogen compound metabolic process | 34 out of 54 genes, 63.0% | 0.01454 |
| metabolic process | 47 out of 54 genes, 87.0% | 0.01676 |
| primary metabolic process | 44 out of 54 genes, 81.5% | 0.0192 |
| organic substance metabolic process | 45 out of 54 genes, 83.3% | 0.02231 |
| organic cyclic compound metabolic process | 32 out of 54 genes, 59.3% | 0.03169 |
| heterocycle metabolic process | 31 out of 54 genes, 57.4% | 0.03385 |
| nucleic acid metabolic process | 28 out of 54 genes, 51.9% | 0.04035 |
| cellular aromatic compound metabolic process | 31 out of 54 genes, 57.4% | 0.0411 |

**Table S14.** The up-regulated genes and down-regulated genes in cell components between BaP and BI-04+BaP.

| Up-regulated EDGs | | Down-regulated EDGs | |
|---|---|---|---|
| GO:0016020 | membrane | GO:0000276 | mitochondrial proton-transporting ATP synthase complex, coupling factor F(o) |
| GO:0005681 | spliceosomal complex | | |
| GO:0000812 | Swr1 complex | GO:0000139 | Golgi membrane |
| | | GO:0000777 | condensed chromosome kinetochore |
| | | GO:0005769 | early endosome |
| | | GO:0030529 | intracellular ribonucleoprotein complex |
| | | GO:0043232 | intracellular non-membrane-bounded organelle |
| | | GO:0019866 | organelle inner membrane |

**Table S15.** The up-regulated genes and down-regulated genes in molecular function between BaP and BI-04+BaP.

| Up-regulated EDGs | | Down-regulated EDGs | |
|---|---|---|---|
| GO:0008270 | zinc ion binding | GO:0004623 | phospholipase A2 activity |
| GO:0016859 | cis-trans isomerase activity | GO:0004661 | protein geranylgeranyltransferase activity |
| GO:0019904 | protein domain specific binding | GO:0005506 | iron ion binding |
| GO:0042802 | identical protein binding | GO:0043168 | phospholipid binding |
| GO:0001071 | nucleic acid binding transcription factor activity | GO:0008135 | translation factor activity, RNA binding |
| GO:0003824 | catalytic activity | GO:0008417 | fucosyltransferase activity |
| GO:0019900 | kinase binding | GO:0008757 | |

| | | | |
|---|---|---|---|
| GO:0032550 | purine ribonucleoside binding | | S-adenosylmethionine-dependent methyltransferase activity |
| GO:0036094 | small molecule binding | GO:0016702 | oxidoreductase activity |
| | | GO:0016811 | hydrolase activity |

**Table S16.** The up-regulated genes and down-regulated genes in biological process between BaP and BI-04+BaP.

| **Up-regulated EDGs** | | **Down-regulated EDGs** | |
|---|---|---|---|
| GO:0006355 | regulation of transcription, DNA-templated | GO:0000723 | telomere maintenance |
| GO:0006366 | transcription from RNA polymerase II promoter | GO:0001501 | skeletal system development |
| GO:0006915 | apoptotic process | GO:0001558 | regulation of cell growth |
| GO:0000377 | RNA splicing | GO:0006468 | protein phosphorylation |
| GO:0008283 | cell proliferation | GO:0007155 | cell adhesion |
| GO:0000122 | negative regulation of transcription from RNA polymerase II promoter | GO:0001666 | response to hypoxia |
| | | GO:0001676 | long-chain fatty acid metabolic process |
| | | GO:0002761 | regulation of myeloid leukocyte differentiation |
| | | GO:0006658 | phosphatidylserine metabolic process |
| | | GO:0006952 | defense response |
